# The Natural Compound Climacostol as a Prodrug Strategy Based on pH Activation for Efficient Delivery of Cytotoxic Small Agents

**DOI:** 10.3389/fchem.2019.00463

**Published:** 2019-06-28

**Authors:** Elisabetta Catalani, Federico Buonanno, Gabriele Lupidi, Silvia Bongiorni, Riccardo Belardi, Silvia Zecchini, Matteo Giovarelli, Marco Coazzoli, Clara De Palma, Cristiana Perrotta, Emilio Clementi, Giorgio Prantera, Enrico Marcantoni, Claudio Ortenzi, Anna Maria Fausto, Simona Picchietti, Davide Cervia

**Affiliations:** ^1^Department for Innovation in Biological, Agro-Food and Forest Systems, Università degli Studi della Tuscia, Viterbo, Italy; ^2^Laboratory of Protistology and Biology Education, Department of Education, Cultural Heritage and Tourism, Università degli Studi di Macerata, Macerata, Italy; ^3^School of Sciences and Technologies, Section of Chemistry, Università degli Studi di Camerino, Camerino, Italy; ^4^Department of Ecological and Biological Sciences, Università degli Studi della Tuscia, Viterbo, Italy; ^5^Department of Biomedical and Clinical Sciences “Luigi Sacco” (DIBIC), Università degli Studi di Milano, Milan, Italy; ^6^Unit of Clinical Pharmacology, University Hospital “Luigi Sacco”-ASST Fatebenefratelli Sacco, Milan, Italy; ^7^Scientific Institute IRCCS “Eugenio Medea”, Bosisio Parini, Italy

**Keywords:** small natural agents, ciliated protists, Wittig olefination, pH-activation, cytotoxic compound, apoptosis, prodrug strategy

## Abstract

We synthesized and characterized MOMO as a new small molecule analog of the cytotoxic natural product climacostol efficiently activated in mild extracellular acidosis. The synthesis of MOMO had a key step in the Wittig olefination for the construction of the carbon-carbon double bond in the alkenyl moiety of climacostol. The possibility of obtaining the target (*Z*)-alkenyl MOMO derivative in very good yield and without presence of the less active (*E*)-diastereomer was favored from the methoxymethyl ether (MOM)-protecting group of hydroxyl functions in aromatic ring of climacostol aldehyde intermediate. Of interest, the easy removal of MOM-protecting group in a weakly acidic environment allowed us to obtain a great quantity of climacostol in biologically active (Z)-configuration. Results obtained in free-living ciliates that share the same micro-environment of the climacostol natural producer *Climacostomum virens* demonstrated that MOMO is well-tolerated in a physiological environment, while its cytotoxicity is rapidly and efficiently triggered at pH 6.3. In addition, the cytostatic vs. cytotoxic effects of acidified-MOMO can be modulated in a dose-dependent manner. In mouse melanoma cells, MOMO displayed a marked pH-sensitivity since its cytotoxic and apoptotic effects become evident only in mild extracellular acidosis. Data also suggested MOMO being preferentially activated in the unique extra-acidic microenvironment that characterizes tumoural cells. Finally, the use of the model organism *Drosophila melanogaster* fed with an acidic diet supported the efficient activity and oral delivery of MOMO molecule *in vivo*. MOMO affected oviposition of mating adults and larvae eclosion. Reduced survival of flies was due to lethality during the larval stages while emerging larvae retained their ability to develop into adults. Interestingly, the gut of eclosed larvae exhibited an extended damage (cell death by apoptosis) and the brain tissue was also affected (reduced mitosis), demonstrating that orally activated MOMO efficiently targets different tissues of the developing fly. These results provided a proof-of-concept study on the pH-dependence of MOMO effects. In this respect, MOM-protection emerges as a potential prodrug strategy which deserves to be further investigated for the generation of efficient pH-sensitive small organic molecules as pharmacologically active cytotoxic compounds.

## Introduction

In recent years, small molecules (molecular weight of <900 Daltons) with well-defined chemical structures and strong biological properties have spurred research aimed at developing new therapeutic agents (Gurevich and Gurevich, 2014). There are indeed several benefits associated with the small molecules that make them more efficient in the field of human health mainly in proliferative, infectious, or neurodegenerative disorders (Gurevich and Gurevich, 2014; Dhanak et al., 2017; Cheng et al., 2018; Huck et al., 2018; Schiavone and Trabace, 2018). Small organic molecules from nature, isolated and exploited from plants, animals and microorganisms, have showed a great translational potential (Nobili et al., 2009; Harvey et al., 2015). Among the natural compounds isolated from aquatic eukaryotic microorganisms (Catalani et al., 2016), climacostol [(Z)-5-(non-2-en-1-yl)benzene-1,3-diol] is a resorcinolic lipid physiologically produced by the freshwater ciliated protozoan *Climacostomum virens* for chemical defense against unicellular and multi-cellular predators (Masaki et al., 1999; Miyake et al., 2003). We have described the antimicrobial activity of climacostol and its synthetic analogs as well as their cytotoxic and pro-apoptotic effects in multiple tumoural cells (Buonanno et al., 2008, 2019; Fiorini et al., 2010; Petrelli et al., 2012; Quassinti et al., 2013; Catalani et al., 2016; Perrotta et al., 2016; Zecchini et al., 2019). Both *in vitro* and *in vivo* evidence demonstrated that climacostol inhibits efficiently the viability/proliferation of mouse melanoma cells, induces a persistent inhibition of tumor growth and improves the survival of transplanted mice thus triggering the cell death process as a result of DNA damage and apoptosis (Catalani et al., 2016; Perrotta et al., 2016). Recently we also showed that climacostol exerts a marked and sustained accumulation of autophagosomes in tumors as the result of dysfunctional autophagic degradation (Zecchini et al., 2019). The up-regulation of p53 system is at the molecular crossroad regulating both the anti-autophagic action of climacostol and its role in the induction of apoptosis (Zecchini et al., 2019). In agreement with the promising paradigm of dual targeting of autophagy and apoptosis in cancer therapy our findings suggest the efficacy of climacostol favoring the consideration of this bioactive small molecule in drug discovery and development.

Cancer tissue often shows low pO_2_ values and an acidic extracellular pH (median pH typically falls below 7.0) in both human and rodent *in vivo* tumors (Wike-Hooley et al., 1984; Tannock and Rotin, 1989; Calcinotto et al., 2012; Chen et al., 2014). Although extracellular acidosis is considered as a toxic consequence of fermentation that is detrimental to cells, it is also recognized as a cytoprotective response, likely maintaining mitochondrial function and cell viability (Khacho et al., 2014), through various anti-inflammatory, anti-oxidant, and anti-apoptotic mechanisms which limit the damage of tumoural tissues (Stubbs et al., 2000; Swietach et al., 2014; Swenson, 2016; Huber et al., 2017; Riemann et al., 2017; Viklund et al., 2017; Damgaci et al., 2018). Besides its role in promoting cancer growth and metastasis, the acidic tumor environment represents a chemical barrier for many chemotherapeutics thus inducing resistance (Stubbs et al., 2000; Huber et al., 2017; Viklund et al., 2017; Damgaci et al., 2018). Growing evidence for the importance of pH in cancer biology has solicited different attractive strategies for therapy including the use of pH-responsive compounds that display increased cytotoxicity at low pH (Adams et al., 2000; Flowers et al., 2003; Wong et al., 2005; Valiahdi et al., 2013; Swietach et al., 2014; Burns et al., 2016; Wang et al., 2018; Sharma et al., 2019).

Similar to healthy tissues exposed to adverse side effects of chemotherapy drugs, *Climacostomum* shares with other toxic ciliates the necessity to avoid or limit autotoxicity that could result by the exposition to its own toxin (Miyake et al., 2001; Buonanno and Ortenzi, 2010; Buonanno et al., 2012, 2014, 2017b). As many multicellular poisonous organisms equipped with specialized organs for safely storing venoms, *Climacostomum* and other toxic ciliates have chosen to store self-toxic compounds in specialized ejectable membrane-bound organelles generally called extrusomes (Wolfe, 2000; Miyake et al., 2001, 2003; Terazima and Harumoto, 2004). In addition to the adoption of this “physical” shield and to further limit autotoxicity in the cytoplasmic compartment, the ciliate *Pseudokeronopsis erythrina* has chosen to synthesize their chemical weapons as sulfonated (not-toxic) molecules prior to store them as not-sulfonated (toxic) molecules into the extrusomes (Anesi et al., 2016; Buonanno et al., 2017a). In this work, we take the self-protection strategy evolved by *Pseudokeronopsis* as a suggestion to allow a safer and target-specific use of climacostol.

**Figure 1:**
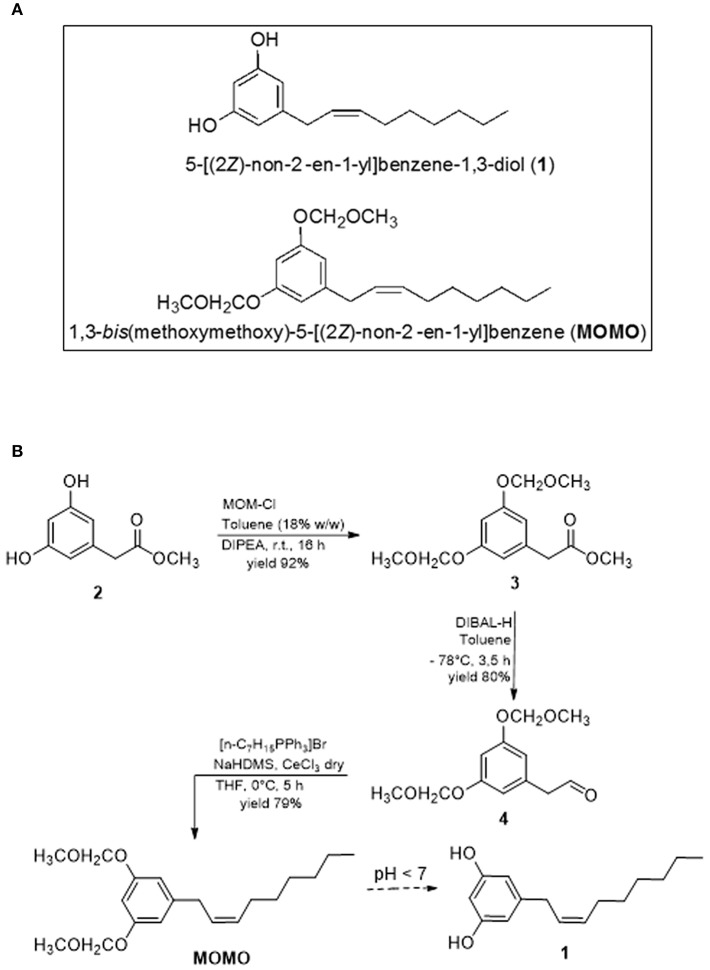
**(A)** Molecular structures of climacostol (**1**) and **MOMO**. **(B)** Total synthesis of MOMO.

We have reported an innovative diastereoselective chemical synthesis of climacostol to obtain a compound more effective than the natural product (Fiorini et al., 2010). The importance of (*Z)*-configuration with regard to the hydrocarbon chain of climacostol is crucial for its biological activity. Since the natural product is a mixture containing the less biologically active (*E)*-diastereomer, a series of synthetic strategies have been studied in the last years to obtain the (*Z)*-configuration. The reduction of the corresponding intermediate alkyne to the double bond is the original synthetic route in the synthesis of bioactive alkenylphenols such as climacostol (Masaki et al., 1999). However, stereoselective synthesis in the corresponding (*Z)*-diastereomer is still challenging in these metal catalyzed reductions, especially when there are problems of shifting double bonds. Moreover, the most used metals exhibit low alkene selectivity due to their high efficiency in promoting further reduction into alkanes, unless high toxic additives are incorporated (Nikolaev et al., 2009; Chung et al., 2013). Although the synthesis improved with the development of new metal nanoparticles catalysts (Sharma and Sharma, 2016; Pedone et al., 2017), the reductions catalyzed by metal nanoparticles tend to aggregate over time, lowering the efficiency of their activities (Chan et al., 2014). The Wittig olefination has thus been chosen as a key step in the construction of the carbon-carbon double bond in the alkenyl moiety of climacostol. In fact, the Wittig reaction is a versatile and reliable method for the production of substituted alkenes due to easy application and good yields of the desired products. The stereoselectivity of the Wittig reaction depends on the substrates (Depré et al., 2017), and all the synthesis of climacostol using the (*Z)*-selective Wittig reaction revealed the presence of the (*E)*-diastereomer (Abe and Mori, 2001). The importance of protection and deprotection of hydroxyl groups in the synthesis of biologically active small molecules (Cappa et al., 1999) helped us to solve the problem of the low quantity of unwanted (*E)*-isomer. The choice of phenol group's protection necessary during the synthesis was particularly interesting in avoiding isomerization of the final carbon-carbon double bond. Only the methoxymethyl ether (MOM) protecting group allowed us to efficiently synthesize climacostol in (*Z)*-configuration biologically active (Fiorini et al., 2010). In particular, screening of mild conditions in phenol MOM-protected allowed us to develop slightly acidic environment (pH around 6.5) able to successfully afford target resorcinol-based climacostol in quantitative yield (Fiorini et al., 2010; Buonanno et al., 2019). This suggested to directly use the MOM-protected molecule (hereafter called MOMO) (Figure 1A) in order to test the ability of pH changes to remove MOM and, possibly, restore the cytotoxic activity of the native molecule climacostol. There are very few examples of MOM-derivative compounds for which their biological activities have been studied (Rowley et al., 1997; Mclennan et al., 2008; Bischoff et al., 2014; Imaoka et al., 2018). To date no small organic molecules containing MOM have been reported as cytotoxic prodrugs.

Here we report on the synthesis and characterization of MOMO to yield a picture of its biological behavior, since the use of pH to control the response of a molecule is of great interest in the biomedical field. In particular, we provide here a proof-of-concept study on the pH-dependence of MOMO effects using different *in vitro* and *in vivo* systems with the aim to identify a new prodrug strategy for the generation of efficient pH-sensitive small organic molecules as pharmacologically active cytotoxic compounds.

## Materials and Methods

### Compounds

Chemically synthesized climacostol (C_15_H_22_O_2_, 5-[(2*Z*)-non-2-en-1-yl]benzene-1,3-diol) was obtained as previously described (Fiorini et al., 2010; Perrotta et al., 2016) and MOMO (C_19_H_30_O_4_, 1,3-*bis*(methoxymethoxy)-5-[(2*Z*)-non-2-en-1-yl]benzene) synthesis was detailed below and in the Results section. Compounds were dissolved in absolute ethanol at 10 mg/ml stock, and stored in the dark at −20°C until use. The stock solutions were diluted in culture medium or diet for ciliate/mammalian cell and Drosophila assays, respectively. Where not indicated, the other reagents were purchased from Sigma-Aldrich (Saint Louis, MO, USA).

### Synthesis of MOMO

#### Chloromethyl Methyl Ether as a Solution in Toluene

A three-neck 500 ml flask fitted with a thermometer, reflux condenser, and addition funnel was charged with dimethoxymethane (DMM, 44.25 ml, 0.50 mol, 1 equiv), toluene (133 ml, 3 volumes), and Zn(OAc)_2_ (9.2 mg, 0.01%). Acetyl chloride (35.5 ml, 0.50 mol, 1 equiv) was placed in the addition funnel, and was then introduced into the reaction mixture at a constant rate over 5 min. The Zn(OAc)_2_ dissolved shortly after addition of the AcCl was started. During the next 15 min, the reaction mixture warmed slowly to 45°C, and then cooled to ambient temperature over 3 h, at which time analysis of an aliquot of the reaction mixture by Nuclear Magnetic Resonance (NMR) indicated complete consumption of DMM. Solutions of MOM-Cl in toluene prepared using this stoichiometry have a density of 0.91 g/ml, are approximately 2.1 M (18% w/w), and are stable for months if adequately sealed. ^1^H NMR (CDCl_3_): δ 5.28 (s, 2H, MOM-Cl), 3.49 (s, 3H, MeOAc), 3.64 (s, 3H, MOM-Cl), 2.02 (s, 3H, MeOAc).

#### Methyl 2-[3,5-Di(methoxymethoxy)phenyl]acetate (3)

To a stirred solution of methyl (3,5-dihydroxyphenyl)acetate (**2**, 0.50 g, 2.94 mmol) in toluene (60 ml) were added *i*-Pr_2_NEt (20 ml, 11.5 mmol) and a 18% w/w solution of chloromethyl methyl ether in toluene (2.1 M, 5.6 ml, 11.76 mmol) dropwise at 0°C under N_2_. The mixture was warmed to r.t. and stirred overnight. CH_2_Cl_2_ was removed in vacuum, the residue taken up in Et_2_O (45 ml), washed with 10% aq HCl (2 × 30 ml), H_2_O (2 × 30 ml), aq 10% NaOH (2 × 30 ml), and brine (45 ml), dried (MgSO_4_), and concentrated at reduced pressure. The residue was purified by silica gel column chromatography (hexanes–EtOAc, 8:2) to give the title compound (0.68 g, 86%) as a colorless oil. IR (neat): 3005, 1730, 1605, 1200, 840 cm^−1^. ^1^ H NMR (400 MHz, CDCl_3_): δ = 3.40 (s, 6 H), 3.62 (s, 2 H), 3.69 (s, 3 H), 5.15 (s, 4 H), 6.60–6.65 (m, 3 Arom). ^13^C NMR (100 MHz, CDCl_3_): δ = 41.0, 51.8, 57.2, 92.3, 98.7, 105.5, 139.0, 157.9, 172.6. MS (EI): *m/z* = 270 [M^+^], 238, 211, 121, 45 (100). Anal. Calcd for C_13_H_18_O_6_: C, 57.77; H, 6.71. Found: C, 57.75; H, 6.70.

#### 2-[3,5-Di(Methoxymethoxy)phenyl]acetaldehyde (4)

A solution of DIBAL-H in toluene (1.0 M, 2.21 ml, 2.21 mmol) was added to a stirred and cooled solution of 3 (0.50 g, 1.85 mmol) in dry toluene (10 ml) at −78°C under N_2_. The reaction mixture was allowed to warm to −60°C while stirring for 3 h, then cooled to −78°C again, and afterwards quenched with MeOH (5 ml). The mixture was filtered through Celite, and the resulting solid was washed with Et_2_O (75 ml). The filtrate and washings were successively washed with H_2_O (2 × 20 ml) and brine (2 × 20 ml), dried (MgSO_4_), and concentrated under reduced pressure. The residue was purified by column chromatography on silica gel (EtOAc–hexanes, 8:2) to give the title compound **4** (0.43 g, 96%) as a pale yellow oil. IR (neat): 3006, 2822, 1735, 1598, 1190, 880 cm^−1^. ^1^H NMR (200 MHz, CDCl_3_): δ = 3.60 (d, *J* = 2.1 Hz, 2 H), 5.75 (s, 4 H), 6.30–6.35 (m, 3 Arom), 9.57 (d, *J* = 2.1 Hz, 1 H). ^13^C NMR (50 MHz, CDCl_3_): δ = 52.6, 56.6, 92.7, 99.3, 104.6, 138.7, 158.6, 200.0. MS (EI): *m/z* = 240 [M^+^], 180, 152, 123, 45 (100). Anal. Calcd for C_12_H_16_O_5_: C, 59.99; H, 6.71. Found: C, 59.98; H, 6.68.

#### n-Heptyltriphenylphosphonium Bromide

To a solution of 1-bromoheptane (25 mmol) in toluene (50 ml) was added Ph_3_P (7.20 g, 27.5 mmol). After refluxing for 48 h, the reaction mixture was cooled to r.t. and the solvent was removed under reduced pressure. The crude product was dissolved in CH_2_Cl_2_ (15 ml), then added dropwise to Et_2_O (75 ml). After stirring for 1 h, the precipitate was filtered and dried under vacuum affording the title compound in pure form (9.92 g, 92%); white crystals; mp 165°C. IR (neat): 3050, 1424, 1265, 895 cm^−1^. ^1^H NMR (400 MHz, CDCl_3_): δ = 0.80 (t, *J* = 6.7 Hz, 3 H), 1.15– 1.21 (m, 8 H), 1.59–1.61 (m, 2 H), 3.80–3.86 (m, 2 H), 7.60–7.65 (m, 15 Arom), 9.57 (d, *J* = 2.1 Hz, 1 H). ^13^C NMR (100 MHz, CDCl_3_): δ = 14.5, 22.0, 22.9, 28.7, 30.5, 31.1, 31.7, 119.0, 130.7, 133.9, 134.9.

#### (Z)-1-[3,5-Dimethoxymethoxy)phenyl]non-2-ene (MOMO)

To a suspension of n-heptyltriphenylphosphonium bromide, previously dried by three azeotropic distillations with anhydrous benzene, (1.92 g, 4.34 mmol) in anhydrous THF (20 ml) was added a solution of sodium hexamethyldisilazide (NaHDMS) in THF (1.0 M, 4.64 ml, 4.64 mmol) at 0°C, and the mixture was stirred at r.t. for 1 h. A solution of **4** (0.30 g, 1.24 mmol) in THF (15 ml) was added to the ylide at −10°C and the resulting mixture was stirred at 0°C for 4 h, and finally stirred at r.t. for 1 h. Sat. aq NH_4_Cl (10 ml) was added to the mixture and extracted with EtOAc (3 × 20 ml). The combined organic phases were washed with brine (2 × 20 ml) and dried (MgSO_4_). Filtration and concentration of the mixture under reduced pressure gave the crude material, which was purified by chromatography on silica gel (EtOAc–hexanes, 1:9) to give 0.35 g (90%) of **MOMO** as a colorless oil. IR (neat): 3003, 1590, 1155, 885 cm^−1^. ^1^H NMR (200 MHz, CDCl_3_): δ = 0.92 (t, *J* = 6.6 Hz, 3H), 1.26-1.42 (m, 8H), 2.14-2.21 (m, 2H), 3.30 (d, *J* = 5.9 Hz, 2H), 3.46 (s, 6H), 5.15 (s, 4H), 5.52–5.61 (m, 2H), 6.65 (d, *J* = 2.2 Hz, 1H Arom.), 6.71 (d, *J* = 2.1 Hz, 2H Arom.). ^13^C NMR (50 MHz, CDCl_3_): δ = 14.3, 22.9, 27.5, 29.3, 29.9, 32.3, 33.9, 56.2, 94.6, 102.5, 110.0, 127.7, 131.6, 144.0, 158.6. MS (EI): *m/z* = 322 [M^+^], 245, 212, 137, 123, 45 (100). Anal. Calcd for C_19_H_30_O_4_: C, 70.77; H, 9.38. Found: C, 70.76; H, 9.37.

### Spectroscopy

All NMR spectra were acquired by Varian 400 or Varian Gemini 200 Spectrometers using standard NMR tubes at 298 K, and operating at 400 or 200 MHz for ^1^H and at 100 or 50 MHz for ^13^C. Residual protic solvent CHCl_3_ (δ_H_ = 7.26) was used as the internal reference, and ^13^C NMR spectra were recorded using the central resonance of CDCl_3_ (δ_C_ = 77.0) as the internal reference. Spectrophotometry was performed by a Fourier-Transform InfraRed (FTIR) spectrometer Perkin-Elmer UATR-2 system. Mass spectrum was recorded on an Agilent 5,988 gas-chromatograph with a mass-selective detector MSD HP 5,790 MS, utilizing electron ionization (EI) at an ionizing energy of 70 eV. A fused silica column (30 m × 0.25 mm HP-5; cross-linked 5% PhMe siloxane, 0.10 μm film thickness) was used with helium carrier flow of 30 ml/min. The temperature of the column was varied, after a delay of 3 min from the injection, from 65 to 300°C with a slope of 15°C min^−1^.

### Ciliated Protists Assays

#### Cultures

*Euplotes aediculatus* clone EA-III (Buonanno et al., 2017b), *Euplotes eurystomus* (collected in Macerata, Italy), *Paramecium multimicronucleatum* clone TL-2 (Buonanno et al., 2017a), and *Paramecium tetraurelia* stock 51 (Buonanno et al., 2013) were cultured in Synthetic Medium for Blepharisma (SMB) (1.5 mM NaCl, 0.05 mM KCl, 0.4 mM CaCl_2_, 0.05 mM MgCl_2_, 0.05 mM MgSO_4_, 2 mM Na-phosphate buffer at pH 6.8, 2 μM EDTA). Cells were fed with the flagellate *Chlorogonium elongatum*, cultivated as previously described (Buonanno, 2005), or in Jaworski's Medium (JM) solution. *Spirostomum ambiguum* stock Pol-5 (Buonanno et al., 2012) and *Spirostomum teres* stock Pol-1 were cultured in bacterized culture medium as previously described (Buonanno and Ortenzi, 2010).

#### Toxicity

Cytotoxicity was evaluated in triplicate samples of 10 ciliate cells placed in depression slides with 250 μl of physiologic SMB (pH 6.8) or slight acidic SMB (pH 6.3, obtained by slowly adding, with stirring, 1 N HCl), containing vehicle or increasing concentrations of toxins (from 0.5 to 20 μg/ml) for 24 h. The number of surviving ciliates (normal morphology and locomotion) was counted and the median lethal concentrations (LC_50_) of treatments was estimated (Buonanno, 2009). To analyse the cytotoxic effects of a sublethal concentration of MOMO on growing and reproduction of ciliates, single specimens of *E. aediculatus, E. eurystomus*, or *P. multimicronucleatum* were fed with the flagellate *C. elongatum* and placed in 250 μl of different solutions prepared with MOMO (0.5 μg/ml) in slight acidic SMB (pH 6.3) or physiologic SMB. Cells were also placed in both acidic SMB or SMB as control. The experiment was conducted in 9 replicates for each single species and solutions. The cells were analyzed after 1 h and every 24, 48, 72, and 96 h. The induction of necrosis was assessed by light microscopy analyzing about 100 cells of *E. aediculatus, E. eurystomus, P. multimicronucleatum, P. tetraurelia, S. ambiguum*, and *S. teres*, treated with 5 μg/ml of MOMO for 20 min, in both physiologic and acidic SMB.

### Mammalian Cell Assays

#### Cell Cultures

Murine melanoma B16-F10 cells, C_2_C_12_ mouse myoblasts and NIH/3T3 mouse fibroblasts (Bizzozero et al., 2014; Cazzato et al., 2014; Assi et al., 2015; Basiricò et al., 2015; Cervia et al., 2016; Perrotta et al., 2016) were cultured in Iscove's or Modified Eagle Medium high glucose supplemented with 10% heat-inactivated fetal bovine serum, glutamine (2 mM), penicillin/streptavidin (100 U/ml), 1% Hepes 1 M (Euroclone), pH 7.2 (physiologic culture medium) (Cervia et al., 2002, 2003, 2005). Cells grown at 37°C in a humidified atmosphere containing 5% CO_2_ (logarithmic growth phase, routine passages every 3 days). Culture media were aseptically adjusted to final pH 7.1, 6.9, 6.7, 6.5, and 6.3 (acidic culture medium) by slowly adding, with stirring, 1 N HCl.

#### 3-(4,5-dimethylthiazol-2-yl)-2,5-diphenyltetrazolium Bromide (MTT) Assay

Mammalian cell viability was determined by MTT assay using published protocols (Cervia et al., 2006, 2007, 2013; Armani et al., 2007; Di Giuseppe et al., 2011; Perrotta et al., 2014). MTT absorbance was quantified spectrophotometrically using a Glomax Multi Detection System microplate reader (Promega, Milano, Italy).

#### Immunofluorescence Detection of Caspase 3 Activity

Using published protocols (Cervia et al., 2016; Cammalleri et al., 2017; Amato et al., 2018), cells cultured in 120-mm coverslips were fixed in 4% paraformaldehyde (PFA) in 0.1 M PBS, pH 7.4, for 10 min and overnight stained with the anti-cleaved-caspase 3 primary antibody at 4°C (Cell Signaling Technology, Danvers, MA, USA), in phosphate buffer saline (PBS) containing 0.5% Triton X-100. Cells were then stained with the appropriate Alexa Fluor secondary antibodies (Life Technologies) for 1 h and cover-slipped in a ProLong Gold Antifade Mountant (Life Technologies), stained with fluorescein phalloidin (cytoskeleton detection) (Life Technologies) and DAPI (nuclei detection). Slides were analyzed using a Zeiss LSM 710 inverted confocal microscope. Images were then optimized for contrast and brightness using Adobe Photoshop.

#### Western Blotting

Using published protocols (Perrotta et al., 2015, 2018; Catalani et al., 2016), cells were homogenized in RIPA lysis buffer, supplemented with a cocktail of protease inhibitors (cOmplete; Roche Diagnostics, Milano, Italy). Equal amounts of proteins were separated by 4–20% SDS-polyacrylamide gel electrophoresis (Criterion TGX Stain-free precast gels and Criterion Cell system; Bio-Rad, Hercules, CA, USA) and transferred onto nitrocellulose membrane using a Bio-Rad Trans-Blot Turbo System. The membranes were probed using the anti-cleaved-caspase 3 primary antibody (Cell Signaling Technology, Danvers, MA, USA). After the incubation with the appropriate horseradish-peroxidase-conjugated secondary antibody (Cell Signaling Technology), bands were visualized using the Clarity Western ECL substrate with a ChemiDoc MP imaging system (Bio-Rad). To monitor for potential artifacts in loading and transfer among samples in different lanes, the blots were routinely treated with the Restore Western Blot Stripping Buffer (ThermoFisher Scientific, Waltham, MA, USA) and re-probed with the goat anti-LDH-A (Santa Cruz Biotechnology, Dallas, TX, USA). Bands were quantified for densitometry using the Bio-Rad Image Lab software.

### *Drosophyla melanogaster* Assays

#### Fly Strain

Oregon-R (Bloomington Drosophila Stock Center, Indiana University Bloomington, IN, USA) was used for all experiments. Flies were cultured for one generation at constant density prior to exposure of eggs or adults.

#### Dietary Conditions

Flies were raised on a standard corn meal agar food (pH 5.5) at 25°C. We prepared our fly food as follows: for 1.2 l of water 100 g of live yeast, 110 g of glucose, 100 g of corn meal and 8 g of agar were added and dissolved in warm water. The mixture was autoclaved and allowed to cool down slowly. The fungicide Nipagin (3 g dissolved in 16 ml of absolute ethanol) was added when the temperature reached ~50°C, and the mixture was then dispensed into vials. For each experiment, wild type flies were reared on 4 ml of either the standard medium (control flies) or MOMO-treated diet (experimental flies). The two MOMO-treated diets contained 100 or 300 μg/ml MOMO; at least 5 vials per dose per experiment were prepared. Standard diet with MOMO vehicle was used as control.

#### Mating Procedure and Developmental Assays

Populations of adult flies (3 days old) were placed in vials (15 females and 10 males) for mating and eggs laying. Vials were visually inspected to ensure copulation was occurring; within 5–30 min, all females were typically paired with males. After 3 days, flies were removed and larval development was checked twice daily. In particular, the number of larvae emerged from food (third instar larvae) at day 5/6 was recorded. Individual eggs were also gently picked after a 24 h copulation of untreated adults using 2% agar plates supplemented with apple juice. We added PBS onto the plate and gently wiped the apple-agar surface with a soft-thin brush. Eggs were then washed in PBS, counted and separated under a stereo microscope in new food vials, i.e., control and experimental diet (50 eggs each), before counting eclosed third instar larvae at day 5/6 and their development into adult. When indicated, females were collected as virgins before housing with males and the individual laid eggs after mating were counted at day 3. For size measurements at least 30 different larvae were considered for each experimental group. Larval length was measured by graph. To determine weight, larvae were frozen and weighed using an ultramicro balance (with high resolution of up to 0.0001 mg).

#### TUNEL and Cleaved-Caspase 3 Staining

Eclosed third instar larvae were fixed in 4% PFA in 0.1 M PBS, pH 7.4, overnight. Each larva was cut at A7 abdominal segment and the anterior part was post-fixed for 5 h at 4°C. Fixed tissues were transferred to 25% sucrose in PBS and stored at 4°C for at least 12 h. Samples were then cut at 15 μm with a cryostat, mounted onto positively charged slides and stored at −20°C until use. TUNEL method (DeadEnd Fluorometric TUNEL System, Promega) was performed according to the manufacturer's instructions and Fluoroshield Mounting Medium containing DAPI (Abcam, Cambridge, UK) was used for nuclei detection. Alternatively, sections were treated for 1 h with 5% bovine serum albumin BSA and 10% of normal goat serum (Life Technologies) in PBS containing 0.5% Triton X-100. Overnight incubation was performed with anti-cleaved-caspase 3 primary antibody (Cell Signaling Technology, Danvers, MA, USA), in PBS containing 0.5% Triton X-100, at 4°C. Sections were then stained with the appropriate Alexa Fluor secondary antibody (Life Technologies) for 1.5 h and finally cover-slipped with Fluoroshield Mounting Medium containing DAPI. TUNEL images were acquired by a Zeiss Axioskop 2 plus microscope equipped with the Axiocam MRC photocamera and the Axiovision software (Carl Zeiss, Oberkochen, Germany). Cleaved-caspase 3 immunostaining images were acquired by a Zeiss LSM 710 inverted confocal microscope. Images were optimized for contrast and brightness using Adobe Photoshop (Adobe Systems, Mountain View, CA, USA).

#### Mitotic Index

To analyse mitotic parameters, brains from the third instar larvae were dissected in hypotonic solution (0,8% sodium citrate) and fixed at room temperature in a freshly prepared mixture of acetic acid/methanol/H_2_O (11:12:2) for 30 s. Single fixed brains were then transferred into small drops of 45% acetic acid on a very clean, dust-free non-siliconized coverslip, for 2 min. A clean slide was lowered onto the coverslip and gentle squashed. Slide was frozen in liquid nitrogen, coverslip was removed with a razor blade and slide immediately immersed in absolute ethanol at −20°C for 15 min. Slides were air-dried and DAPI stained, 0.2 μg/ml in 2x saline sodium citrate (20x: 0.15 M NaCl, 0.015 M sodium citrate) to detect chromatin and mitotic figures (Fabbretti et al., 2016). Mitotic index was defined as the number of mitotic cells per optical field (Volpi et al., 2013). The optical field was the circular area defined by a 100x Zeiss objective/1.30 Plan-NEOFLUAR, using 10x oculars and the Optovar set at 1.25 (Zeiss Axiophot microscope). Every optical field occupied by brain tissue was scored (three brains each slide, at least 50–100 optical fields per slide).

### Statistics

LC_50_ (the median lethal concentration), IC_50_ (the concentration producing half the maximum inhibition), and E_max_ (the maximum effect) were determined by non-linear regression curve analysis of the concentration-effect responses. The difference in sample means of LC_50_ were expressed by the 95% level of confidence, essentially according to the procedure previously described (Buonanno, 2009). Potency values among concentration-response curves were compared by the F-test. Statistical significance of raw data between the groups in each experiment was evaluated using one-way ANOVA followed by the Newman-Keuls post-test (multiple comparisons). Data belonging from different experiments were represented and averaged in the same graph. The GraphPad Prism software package (GraphPad Software, San Diego, CA, USA) was used. The results were expressed as means ± SEM of the indicated n values.

## Results

### Synthesis of MOMO

The key step for the synthesis of MOMO is the (*Z*)-diastereoselective Wittig reaction on intermediate **4** (Figure 1B), that has been developed in the diastereoselective climacostol synthetic strategy (Fiorini et al., 2010), and some of biologically active analogs of climacostol (Buonanno et al., 2019). The possibility of obtaining the target (*Z*)-alkenyl MOMO derivative in very good yield and without presence of the (*E*)-diastereomer is favored from the MOM-protecting group of hydroxyl functions in aromatic ring of aldehyde **4**. Compound **4** was subjected to Wittig reaction with n-heptyltriphenylphosphonium salt, prepared using NaHMDS (Dickschat et al., 2005). The generated ylide reacts fast to produce (*Z*)-olefin, and the side reactions, such as homo-olefination of Wittig reagent, was inhibited by the presence of CeCl_3_.

In the synthesis of climacostol and its analogs, the protection of hydroxyl groups as MOM not only ensured the preparation of the desired (*Z*)-alkenyl intermediate without the presence of (*E*)-diastereomer, but allowed the development of an efficient methodology for the final MOM-deprotection. This occur in mild acidic conditions (pH around 6.5) to free hydroxyl groups without olefin isomerization and in excellent yield (Fiorini et al., 2010; Buonanno et al., 2019). The ability to synthesize the MOMO target compound without the presence of any impurities that could interfere with its biological activity was also crucial. Generally, the MOM group is introduced by treatment of the hydroxyl derivatives with very reactive chloromethyl methyl ether (MOM-Cl). Even if less toxic reagents are available, the MOM-Cl is no more dangerous than other chemicals. However, it is known that MOM-Cl commercially available is typically contaminated with dichloromethyl methyl ether, a by-product of its preparation, which is reported to be highly carcinogenic (Narasaka et al., 1984). For this reason, we attempted a number of methods for the introduction of the MOM groups in **2** which do not rely on the chloride. The methods are all based on the use of CH_2_(OCH_3_)_2_ (dimethoxymethane) with various catalysts (Shyamsundar et al., 2018). Unfortunately, all the methodologies were not satisfactory for our phenol moiety, owing to such drawbacks as very low yield and long reaction time. Also, the catalysts were not easily obtained, expensive, and unrecoverable. Consequently, we utilized a method for the rapid preparation of a solution of MOM-Cl in toluene (18% w/w) by the Zn(OAc)_2_-catalyzed exchange reaction between acetyl chloride and dimethoxymethane (Berliner and Belecki, 2005). This solution does not contain the dichloromethyl methyl ether by-product, it is stable for several months if adequately sealed, it is able to give intermediate **3** in very good yield, and above all the excess reagent is decomposed on workup, thereby minimizing exposure to carcinogenic compounds. The subsequent reduction of **3** with DIBAL-H afforded the desired aldehyde **4** with an excellent yield.

### Spectroscopic Analysis of MOMO

**Figure 2:**
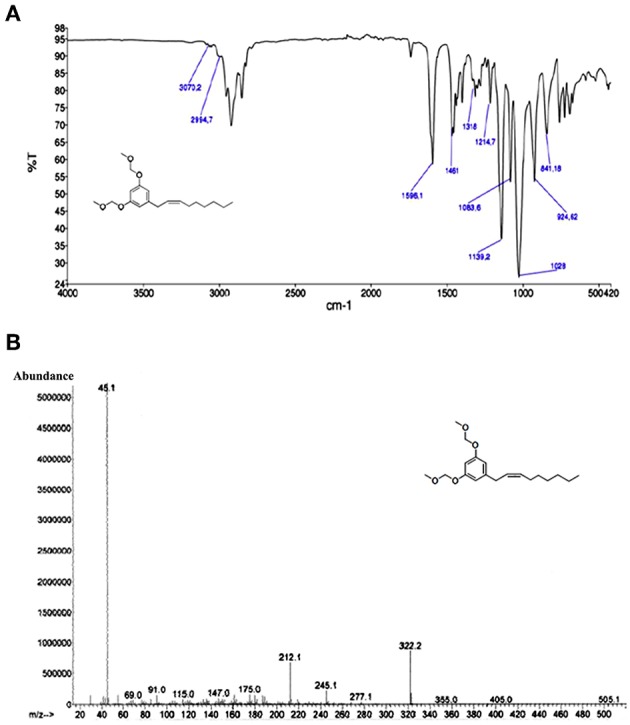
**(A)** FTIR and **(B)** EI-MS spectra of MOMO.

The structure of MOMO was established mainly on the basis of analysis of NMR, FTIR spectroscopic, and mass spectrometry data. Compound MOMO was obtained as colorless oil, and FTIR spectrum displayed the absence of the strong 970 cm^−1^ band for the (*E)*-geometry (Figure 2A) to suggest the only (*Z*)-geometry for the disubstituted double bond (Vanaller et al., 1983). The mass spectrum of this compound (Figure 2B) exhibited a molecular ion [M^+^] peak at *m/z* 322 in the electron ionization mass spectrum (EI-MS), which corresponded to the molecular formula C_19_H_30_O_4_ of MOM-climacostol protected. The presence of a peak base at *m/z* 45 is in agreement with a methoxymethyl moiety, and assignment of the double bond at C-2′ was based on the fragment ion peak observed at *m/z* 212 resulting from MacLafferty rearrangement of aromatic ring, and this was a confirmation that in our conditions the Wittig reaction proceeds without isomerization Δ^2^′, 3′-Δ^1^′, 2′ of the carbon-carbon double bond in the alkenyl chain (Knödler et al., 2007).

**Figure 3:**
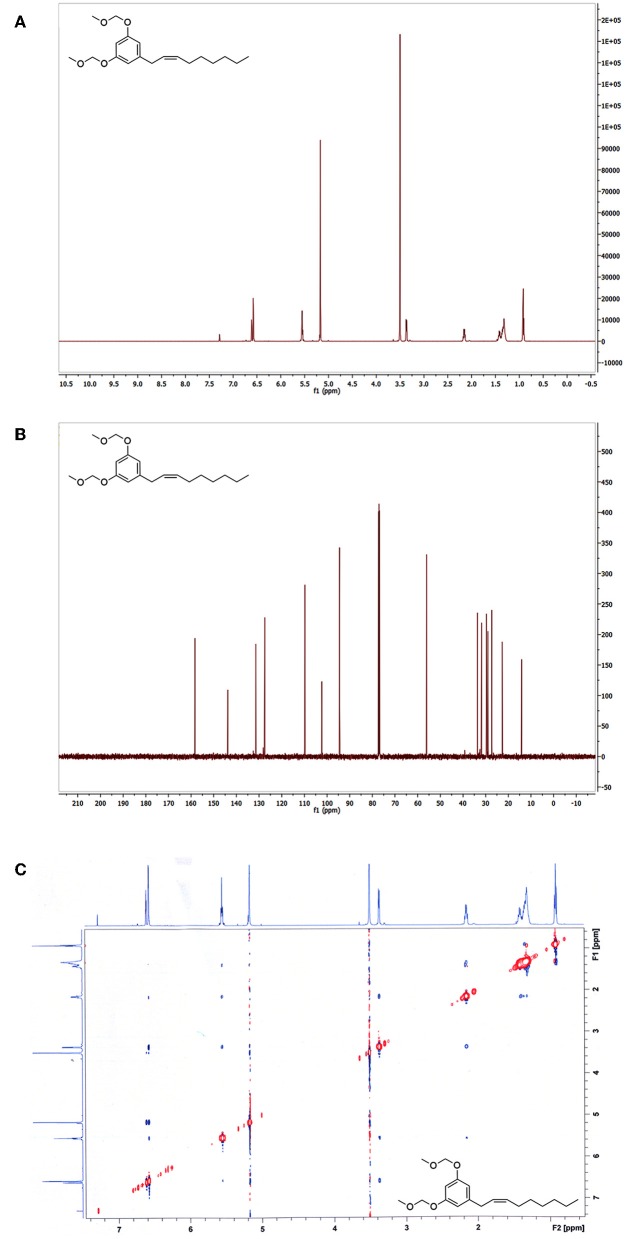
**(A)** ^1^H-NMR, **(B)** ^13^C-NMR, and **(C)** ^1^H-^1^H ROESY spectra of MOMO.

Analysis of the ^1^H NMR spectroscopic data confirmed a pair of aromatic *meta*-coupled protons at δ 6.65 and δ 6.71, as well as proton signals belonging to two methoxyl groups (-OCH_3_) at δ 3.46 and oxymethyl ethers (-OCH_2_O-) at δ 5.15 (Figure 3A). A terminal methyl at δ 0.92, olefinic methines at δ 5.52–5.61 (overlapped), and an allylic methylene at δ 3.30 confirm the presence of an alkenyl chain with the carbon-carbon double bond between C-2′ and C-3′. The assignment of the double bond at C-2′ was also based on the ^13^C NMR (Figure 3B) chemical shift value of the two allylic carbons at δ 33.9 and δ 32.3. It is reported that the chemical shift values of the allylic carbons of (*E*)-diastereomer in alkenylresorcinols are greater than values of (*Z*)-diastereomer (Liu et al., 2009). The presence of a single diastereomer, however, did not permit to assign the configuration of the double bond by comparison with literature data of both diastereomer (Al-Mekhlafi et al., 2012). Also, the proton signals H-2′ and H-3′ in ^1^H NMR spectrum were not well-separated, and measuring the value of the coupling constant of the olefinic protons in the 1D spectrum was difficult. All these experimental observations in ^1^H NMR and ^13^C NMR spectra allowed us to observe the presence of a single diastereomer, but not to solve the configuration (*Z*) or (*E*) of the single diastereomer. Unlike what observed for the climacostol and its analogs, we confirmed MOMO as a product having a (*Z*)-configuration by a ROESY analysis (Crouch et al., 1990). The ^1^H-^1^H ROESY (Rotating from Overhause Effect SpectroscopY) is useful for determining which signals arise from protons that are close to each other in space even if they are not bonded (Kessler et al., 1987), and remarkable cross-peaks were detected between the methylene protons in C-1' and between methylene protons in C-4′ (red circles in Figure 3C). This interesting cross-peaks detected at room temperature can be observed in the (*Z*)-configuration diastereomer, where the protons involved are closer in space than in the (*E*)-diastereomer, for which the cross-peaks should not be observed. This means that the two protons in C-1' are on the same side of the protons in C-4', further demonstrating the importance of MOM-protecting group to obtain the (*Z*)-configuration for the disubstituted double bond of the small molecule MOMO.

### pH-Induced Activation of MOMO in Native Systems

The basic functions of climacostol are to defend the ciliate *Climacostomum virens* from predators or to assist the ciliate carnivorous feeding (Masaki et al., 1999; Miyake et al., 2003), likely reducing the competition for food resources. To assess the cytotoxicity of the prodrug MOMO in native system, the LC_50_ of MOMO (increasing concentrations from 0.5 to 20 μg/ml) in physiologic (pH 6.8) or mild acidic (pH 6.3) SMB was obtained treating four prey (*Paramecium tetraurelia, P. multimicronucleatum, Spirostomum ambiguum*, and *S. teres*) and two competitors (*Euplotes aediculatus* and *E. eurystomus*) of *C. virens* for 24 h. Data were also compared with those previously obtained with native compound climacostol in physiologic SMB (Buonanno et al., 2019). As summarized in Table 1, no toxicity for MOMO was reported in SMB at pH 6.8. Of interest, the cytotoxic effects of MOMO become evident in acidic SMB, comparable with climacostol.

**Table 1:** Median lethal concentrations (LC_50_: μg/ml) of MOMO obtained in six ciliate species.

**Ciliate species**	**MOMO (pH 6.8)**	**MOMO (pH 6.3)**	**Climacostol**
**E*.*aediculatus**	>20	1.31 (0.48−3.58)	0.83 (1.62−1.79)^*^
**E*.*eurystomus**	>20	1.28 (0.10−1.66)	0.70 (0.65−0.76)
**P*.*multimicronucleatum**	>20	2.21 (1.57−3.11)	0.88 (0.18−4.45)^*^
**P*.*tetraurelia**	>20	1.46 (1.04−2.05)	0.90 (0.37−2.19)^*^
**S*.*ambiguum**	>20	2.12 (1.27−3.53)	1.66 (1.43−1.92)^*^
**S*.*teres**	>20	1.90 (1.08−3.34)	1.68 (1.55−1.83)^*^

*Viability was assessed after 24 h incubation with increasing concentrations of MOMO in physiologic SMB (pH = 6.8) and in acidic SMB (pH = 6.3). No sign of cytotoxicity was detected for the solutions of acidic SMB mixed with vehicle control (ethanol 2%; data not shown). The LC_50_ values were obtained by non-linear regression analysis of three independent experiments, with the 95% confidence limits in parenthesis. Data were also compared with those obtained with native compound climacostol in physiologic SMB*.

* *: (Buonanno et al., 2019)*.

**Figure 4:**
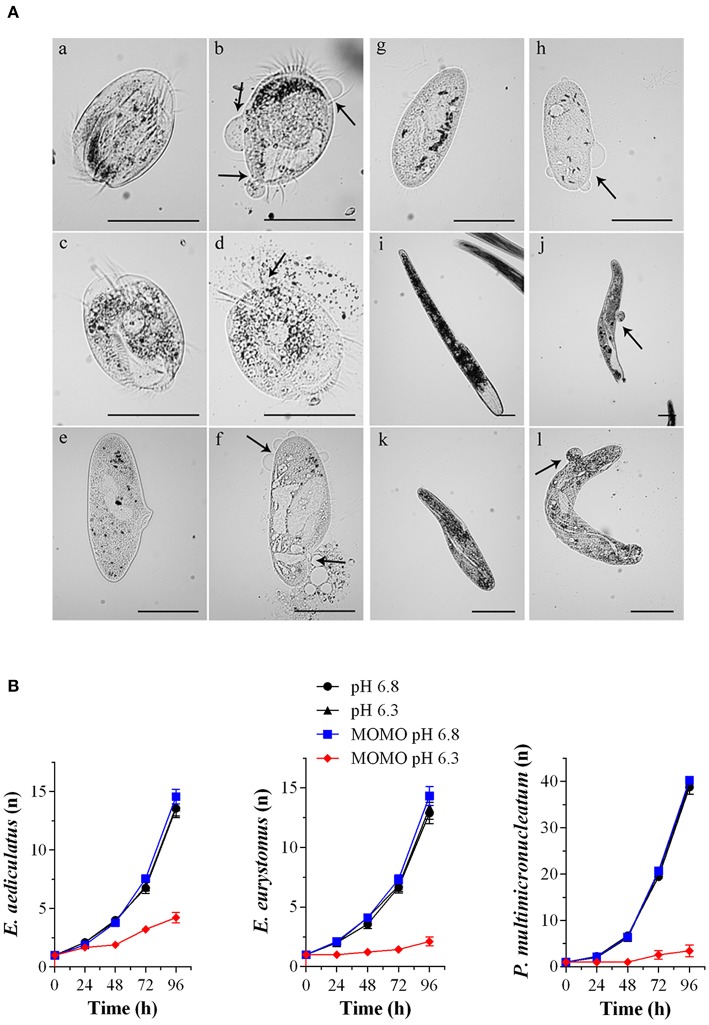
MOMO cytotoxicity in native systems. **(A)** Cell death effects of MOMO (5 μg/ml) in acidic SMB at pH 6.3 (b, d, f, h, j, l) and in physiological SMB (a, c, e, g, i, k) on six species of ciliated protists: *Euplotes aediculatus* (a, b), *Euplotes eurytomus* (c, d), *Paramecium multimicronucleatum* (e, f), *Paramecium tetraurelia* (g, h), *Spirostomum ambiguum* (i, j) and *Spirostomum teres* (k, l). Necrosis was observed only for MOMO solutions in acidic SMB after 20 min for all ciliate used. Arrows indicate plasma membrane swellings or fractures. The images are representative of 10 independent observations. Scale bars = 100 μm. **(B)** Growth curves of *Euplotes aediculatus, Euplotes eurystomus*, and *Paramecium multimicronucleatum* in acidic SMB (pH 6.3) and physiologic SMB (pH 6.8) both in the absence and in the presence of MOMO (0.5 μg/ml). All specimens were fed in order to compare their ability to multiply. Each point (*n* = cell number) is representative of 9 independent experiments.

Accordingly, when the ciliates were acutely treated with 5 μg/ml of MOMO for 20 min in acidic SMB, they showed evident signs of necrosis, a form of cell injury characterized by loss of plasma membrane integrity and uncontrolled release of cellular components into the extracellular space (Figure 4A). Similar results were obtained with climacostol (Buonanno et al., 2019). In contrast, the solution of MOMO resuspended in physiological SMB did not display any toxic effect on all the three species used. No toxicity was detected for acidic SMB or for control vehicle diluted in acidic SMB (data not shown). In addition, to detect potential effects of sublethal concentrations of MOMO in acidic SMB (pH 6.3), specimens of *E. aediculatus, E. eurystomus* and *P. multimicronucleatum* were fed and incubated with MOMO (0.5 μg/ml) in acidic SMB. Cells exposed to MOMO at increasing times showed normal morphology and locomotion with no sign of necrotic processes. They were also compared with those growing in physiological conditions for their fission rate. The results indicated that the proliferative capability of MOMO-treated cells was effectively inhibited (Figure 4B). In contrast, no difference in fission rate was detected for the three species exposed to MOMO in physiological conditions.

Overall, the collected data demonstrate that MOMO is well-tolerated by ciliate species in a physiological environment, while its cytotoxicity (activation) is rapidly and efficiently triggered by mild acidification of the medium. Worth of note, the biological activity (cytostatic vs. cytotoxic) of acidified-MOMO observed in ciliated protists can be modulated in a dose-dependent manner.

### pH-Induced Activation of MOMO in Mammalian Cells

**Figure 5:**
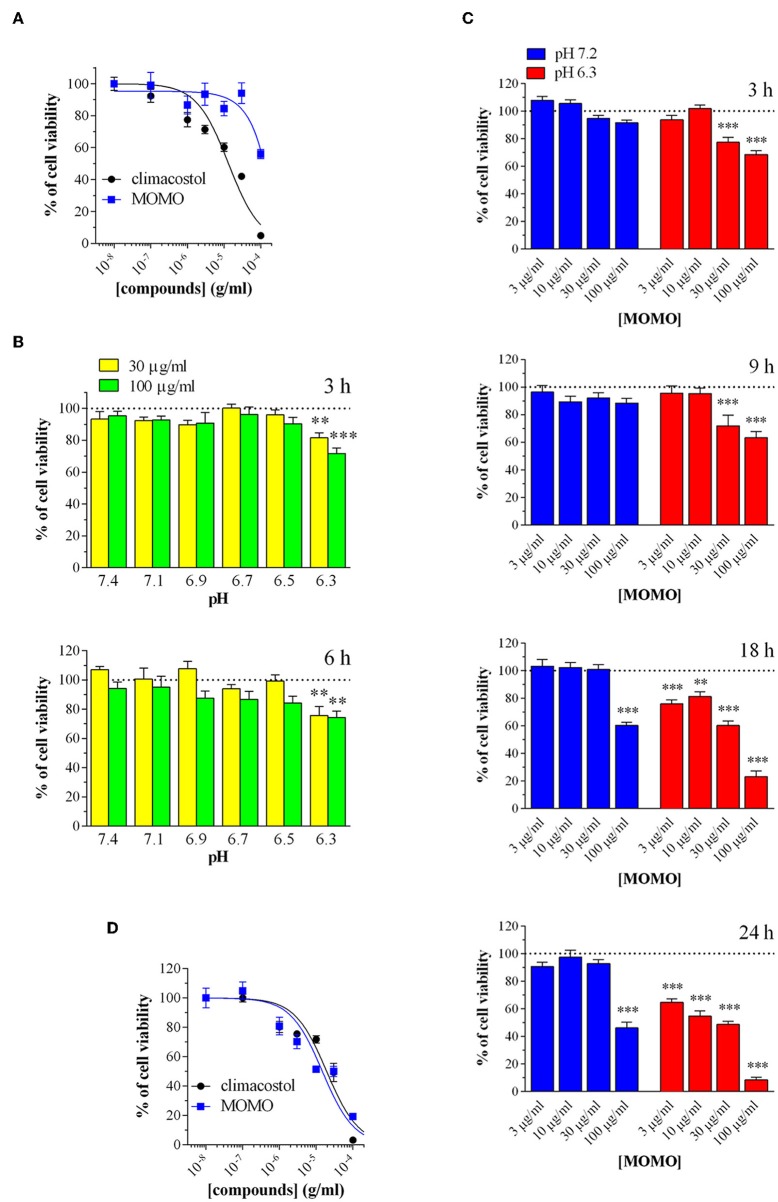
MOMO cytotoxicity in melanoma cells. Mouse B16-F10 cells were treated with increasing concentrations of MOMO and climacostol for **(A)** 24 h or **(D)** 48 h, before MTT assay. The data points are representative of 4–10 independent experiments. MTT assay after B16-F10 cell treatment with: **(B)** 30 and 100 μg/ml MOMO (3 or 6 h incubation) in culture medium ranging from pH 7.1 to 6.3, **(C)** increasing concentrations of MOMO (3, 9, 18, and 24 h incubation) in physiologic (pH 7.2)/acidic (pH 6.3) culture medium. Data are representative of at least 6 independent experiments. ^**^p < 0.001 and ^***^p < 0.0001 relative to the respective control. Results are expressed by setting the absorbance of the reduced MTT in the respective control (vehicle-treated) samples, i.e., absence of compounds, as 100%.

We examined the effects of MOMO on cell viability *in vitro* mouse B16-F10 melanoma cells. Cells were firstly analyzed by MTT assay in physiologic culture medium (pH 7.2) after cell treatment with increasing concentrations of MOMO. In agreement with previous reports (Catalani et al., 2016; Perrotta et al., 2016; Zecchini et al., 2019), the native climacostol, used as a control compound, dose-dependently decreased melanoma cell viability by almost 100% (E_max_) when applied for 24 h (Figure 5A). In contrast, the MTT absorbance was reduced by MOMO (44% inhibition) only at the highest concentration tested, i.e., 100 μg/ml, while the lower concentrations were devoid of activity.

Then, to test if extracellular acidification may effectively activate MOMO molecule, melanoma cell viability was assessed in culture medium ranging from pH 7.1 to 6.3. In particular, cells were incubated in the absence (vehicle) and presence of MOMO for 3 and 6 h. As shown in Figure 5B, short treatments with MOMO at 30 and 100 μg/ml significantly decreased MTT absorbance in cells exposed to pH 6.3 medium, but not to more alkaline medium. The role of pH was then further dissected using increasing concentrations of MOMO at increasing times. In physiologic culture medium (pH 7.2) MOMO partially reduced B16-F10 cell viability only at 100 μg/ml and after long exposures, i.e., 18 and 24 h, while cell incubation in mild acidic medium (pH 6.3) efficiently increased MOMO activity (Figure 5C). Of notice, at 18 and 24 h even 3 μg/ml MOMO (the lower concentration tested) significantly decreased MTT absorbance vs. control. In addition, 100 μg/ml MOMO exerted its maximal effect (almost 100% inhibition) between 18 and 24 h thus indicating that the cytotoxic effect of MOMO is markedly enhanced in acidic extracellular environment. During prolonged cell culture the pH of the medium normally decreased as also indicated by the yellow color of the phenol red. Accordingly, similarly to the native climacostol, MOMO dose-dependently decreased B16-F10 cell viability when applied for 48 h, even in physiologic culture medium (Figure 5D).

**Figure 6:**
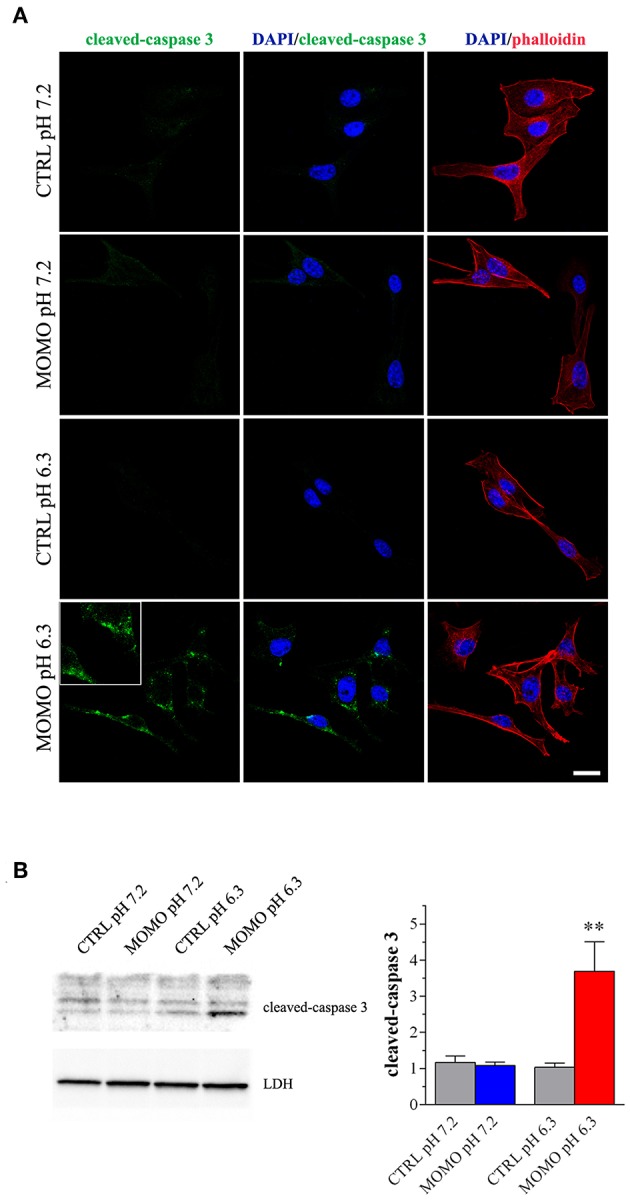
MOMO-induced apoptosis in melanoma cells. Mouse B16-F10 cells were cultured in the presence of 30 μg/ml MOMO or vehicle (CTRL) for 9 h in physiologic (pH 7.2)/acidic (pH 6.3) culture medium. **(A)** Immunofluorescence imaging of cleaved-caspase 3 (punctate green pattern). DAPI (blue) and phalloidin (red) were used for nuclei and cytoskeleton detection, respectively. Inserts represent enlarged image details. Scale bar = 20 μm. **(B)** Western blotting image of cleaved-caspase 3. LDH was used as internal standard. Low panel: densitometric analysis of cleaved-caspase 3. Results are expressed as fold change of CTRL. ^**^p < 0.001 relative to CTRL. Images and data are representative of 6 independent experiments.

Immunostaining with a fluorescently labeled antibody that binds specifically to cleaved-(active) caspase 3, an hallmark of apoptosis, revealed that B16-F10 cells treated with submaximal MOMO (30 μg/ml, 9 h) expressed active caspase 3 when exposed to pH 6.3 but not pH 7.2 medium (Figure 6A). No specific stain was observed in control medium. These results were confirmed by western blot experiments since MOMO-treated melanoma cells in mild acidic culture medium (pH 6.3) showed a significant increase of cleaved-caspase 3 expression when compared to control (Figure 6B); thus demonstrating the pro-apoptotic effects of pH-activated MOMO in tumor cells, similarly to the native climacostol (Buonanno et al., 2008; Perrotta et al., 2016; Zecchini et al., 2019).

**Figure 7:**
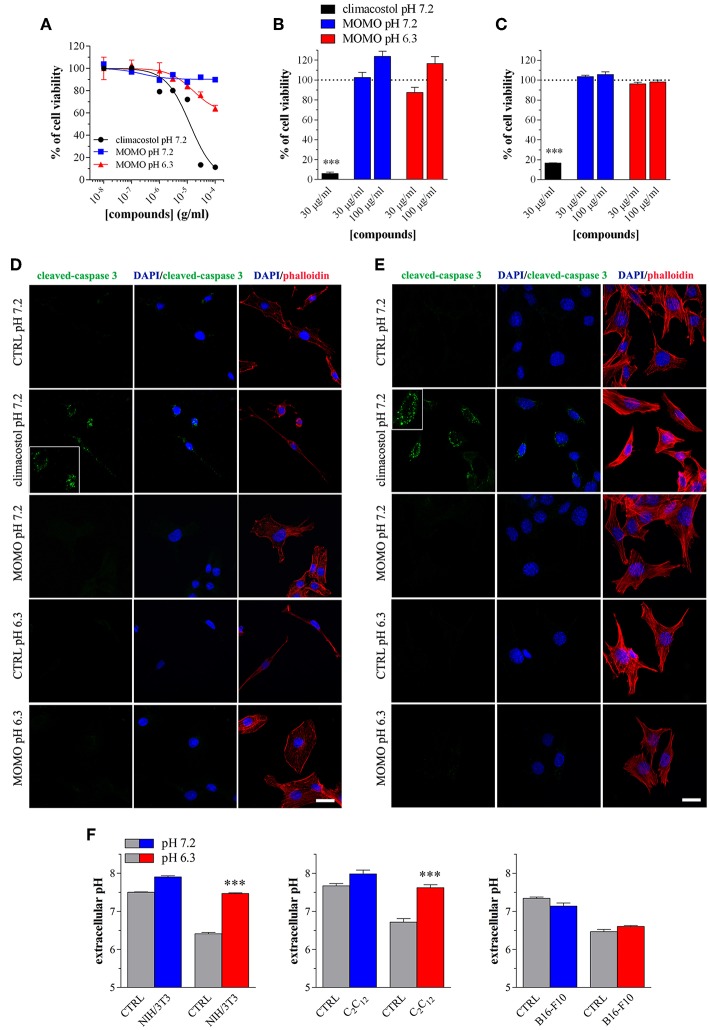
MOMO cytotoxicity/apoptosis in non-transformed mouse cells. **(A)** NIH/3T3 fibroblasts were treated with increasing concentrations of MOMO for 24 h in physiologic (pH 7.2)/acidic (pH 6.3) culture medium, before MTT assay. Climacostol in physiologic medium was used as a reference compound. The data points are representative of 5–6 independent experiments. MTT assay after **(B)** NIH/3T3 fibroblasts or **(C)** C_2_C_12_ myoblasts treatment with 30 and 100 μg/ml MOMO (9 h incubation) in physiologic (pH 7.2)/acidic (pH 6.3) culture medium. Climacostol (30 μg/ml, 9 h, pH 7.2 medium) was used as a reference compound. Data are representative of 4 independent experiments. ^***^p < 0.0001 relative to the respective control. Results are expressed by setting the absorbance of the reduced MTT in the respective control (vehicle-treated) samples, i.e., absence of compounds, as 100%. Immunofluorescence imaging of cleaved-caspase 3 (punctate green pattern) in **(D)** NIH/3T3 and **(E)** C_2_C_12_ cells cultured in the presence of 100 μg/ml MOMO or vehicle (CTRL) for 9 h in physiologic (pH 7.2)/acidic (pH 6.3) culture medium. Climacostol (30 μg/ml, 9 h, pH 7.2 medium) was used as a reference compound. DAPI (blue) and phalloidin (red) were used for nuclei and cytoskeleton detection, respectively. Inserts represent enlarged image details. Scale bar = 20 μm. **(F)** Measurements of pH of physiologic and acidic culture medium after 9 h incubation in the absence (CTRL) and in the presence of NIH/3T3, C_2_C_12_, and B16-F10 cells (plated at the same density in 100 mm-Petri dishes). ^***^p < 0.0001 relative to the respective CTRL.

We also tested the cytotoxic activity of MOMO in immortalized mouse cells of non-tumor origin (non-transformed cells). Climacostol, used as a reference compound, potently and efficiently inhibited the MTT absorbance of NIH/3T3 fibroblast cells cultured for 24 h in physiologic medium (Figure 7A). No effects of MOMO were detected at pH 7.2 whereas, surprisingly, it displayed only a slight efficacy (E_max_ ca. 40%) in mild acidic (pH 6.3) culture medium. Additionally, 9 h after NIH/3T3 cell treatment with 30 and 100 μg/ml MOMO no detectable effects on MTT absorbance were achieved in physiologic and acidic culture medium, while 30 μg/ml climacostol (pH 7.2 medium) robustly decreased cell viability (Figure 7B). Similar results were obtained with C_2_C_12_ myoblast cells (Figure 7C). In addition, NIH/3T3 and C_2_C_12_ cells treated with 100 μg/ml MOMO for 9 h did not express cleaved-caspase 3 immunostaining when exposed to both physiologic and acidic culture medium, while caspase 3 activation was observed in the presence of climacostol (30 μg/ml, 9 h) in physiologic culture medium (Figures 7D,E). Taken together these results indicate the very low cytotoxic/pro-apoptotic activity of MOMO vs. cultured NIH/3T3 and C_2_C_12_ cells.

The simplest explanation may rely on the fact that cells experience different challenges to the low extracellular pH, which depend both on the individual cell and cell type (Salameh et al., 2014). We thus measured the extracellular pH of NIH/3T3, C_2_C_12_ and B16-F10 cells cultured for 9 h in both physiologic and acidic culture medium. As show in Figure 7F, in the presence of NIH/3T3 and C_2_C_12_ cells, but not B16-F10, the acidic culture medium become robustly alkaline and this likely affects (negatively) MOMO activation.

### *In vivo* Toxicity of MOMO in *Drosophila melanogaster*

The fruit fly *D. melanogaster* was then used as it is considered a very potent *in vivo* tool to detect the potential damaging effect of new compounds (Pandey and Nichols, 2011). In the fly life cycle, larvae hatch from eggs and eat continuously, stopping only to molt twice after first instar and second instar stages. During this time, larvae consume nutrients in preparation for pupation. At 5/6 days after egg laying, third instar larvae leave the food and “wander” as they prepare to undergo metamorphosis into the adult fly. Of notice, an acidic diet is the standard practice for maintaining *D. melanogaster* since helps to suppress bacterial growth, increases food palatability and consumption and extends the lifespan compared with flies on either neutral or alkaline pH food (Deshpande et al., 2015). For these reasons Drosophila is a suitable model to test MOMO activated by low pH. In addition, the feeding experiments in Drosophila are important to support the efficient activity and oral delivery of the molecule *in vivo*, as a first step toward evaluating the biological effects of prodrug MOMO in a non-target multicellular organism.

**Figure 8:**
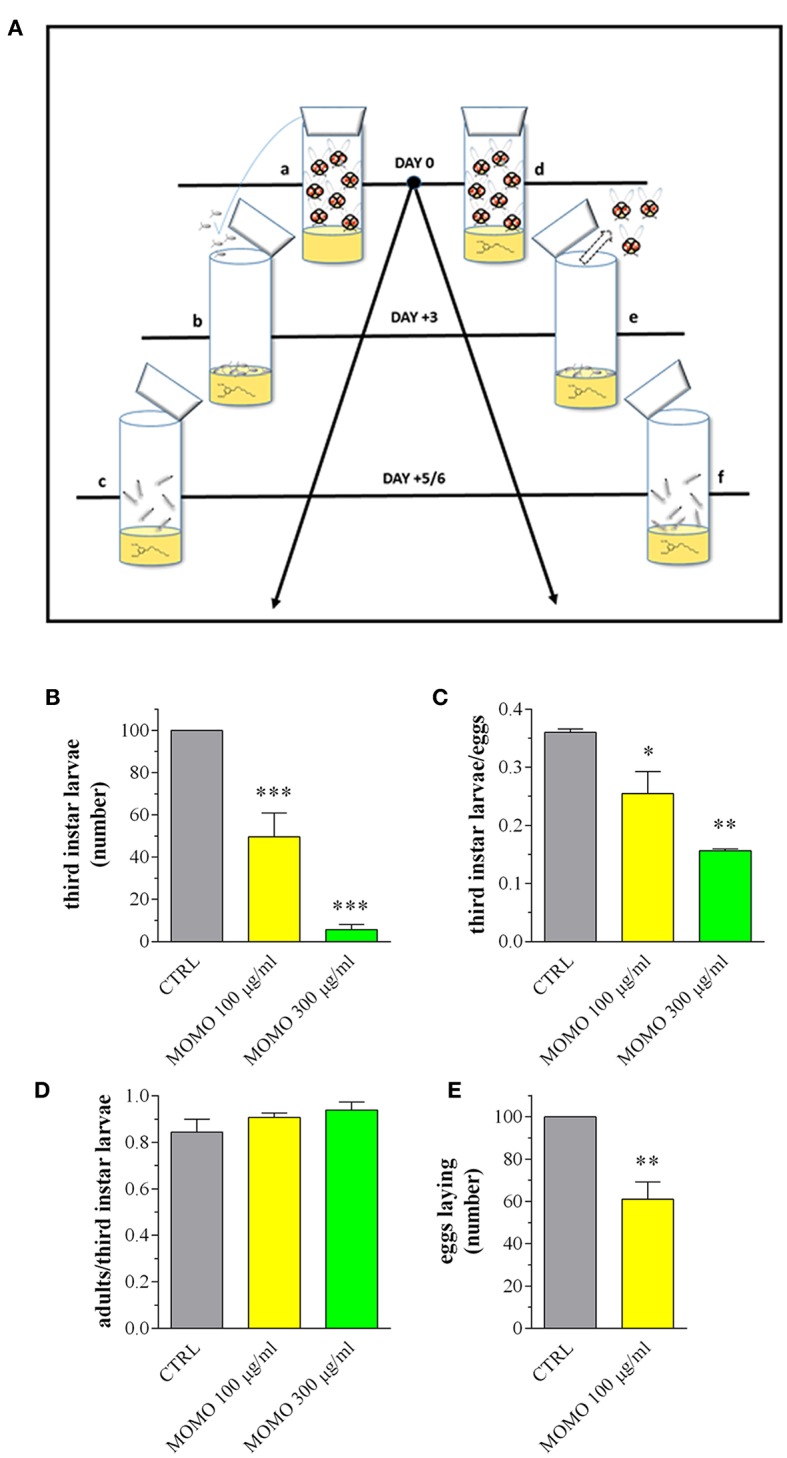
MOMO toxicity in *Drosophila melanogaster*. **(A)** Experimental schedule. a: flies mating and eggs laying on standard food (15 females and 10 males); b: eggs sampling and splitting on new standard food (vehicle) or containing MOMO (100, 300 μg/ml), *n* = 5 per group; c: third instar larvae sampling; d: flies mating and eggs laying (15 females and 10 males) on standard food (vehicle) or containing MOMO (100, 300 μg/ml), *n* = 5 per group; e: adults removing; f: third instar larvae sampling. **(B)** Number of third instar larvae emerging from vials at day 5/6. Results are expressed by setting the control (CTRL, vehicle) as 100%. **(C)** Third instar larvae emerging at day 5/6 from vials containing an equivalent number of eggs (50). Results are expressed by setting the number of eclosed larvae vs. the number of eggs. **(D)** Number of flying adults vs. the number of eclosed third instar larvae. **(E)** Number of laid eggs when females of *Drosophila* were collected as virgins before copulation. Results are expressed by setting the CTRL as 100%. Data are representative of 5 independent experiments. ^*^p < 0.01, ^**^p < 0.001, and ^***^p < 0.0001 relative to CTRL.

We determined if *D. melanogaster* development was affected upon ingestion of MOMO (Figure 8A). Mating flies were placed in vials containing standard Drosophila diet (pH 5.5) mixed with MOMO at increasing concentrations. After eggs laying, flies were removed and larval survival was checked measuring the number of third instar larvae emerged from food. As shown in Figure 8B, third instar larvae number was significantly reduced in both 100 and 300 μg/ml MOMO-treated groups when compared to control (vehicle-treated). Results exhibited a dose-dependent response to treatments as the effect of 300 μg/ml MOMO was more pronounced, thus indicating the efficiency of acidic pH on the biological activation of MOMO molecule. Similar results were obtained counting the number of eclosed larvae emerging from food in vials containing an equivalent number of eggs (Figure 8C). Noteworthy, reduced survival was limited to the larval stages, as the percentage of developed adults, which represents the amount of adults out of the total number of eclosed larvae was not significantly different for flies reared on MOMO food vs. flies reared in control food (Figure 8D). In addition, when females of Drosophila were collected as virgins before copulation, the number of laid eggs in 100 μg/ml MOMO-treated group vs. control was significantly reduced (Figure 8E).

**Figure 9:**
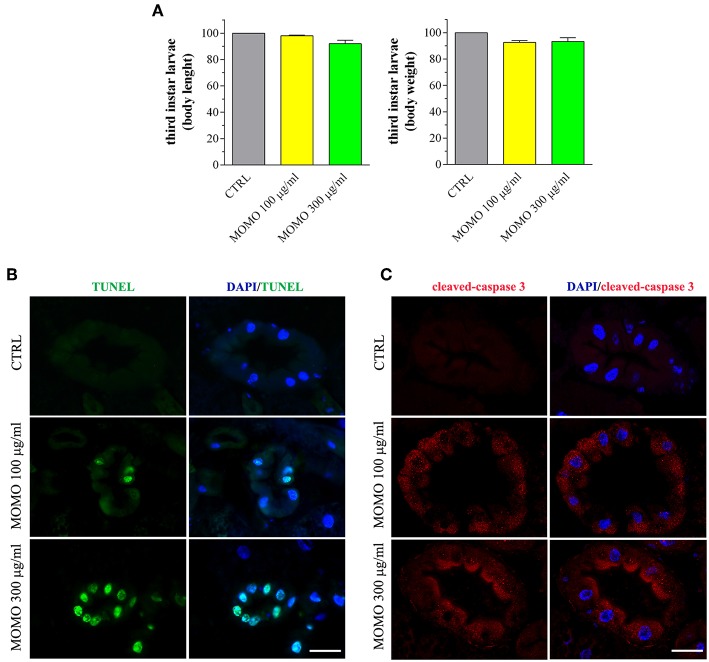
MOMO-induced apoptosis in gut of *Drosophila melanogaster*. Third instar larvae emerging at day 5/6 from vials containing control food (CTRL, vehicle) or supplemented with increased concentrations of MOMO were analyzed. **(A)** Body length and weight. Results are expressed by setting the CTRL as 100%. Data are representative of 5 independent experiments. **(B)** TUNEL and **(C)** cleaved-caspase 3 staining of midgut digestive tract. DAPI (blue) was used for nuclei detection. Data are representative of at least 15 larvae collected in 5 independent experiments. Scale bar = 50 μm.

Third instar larvae were then sampled from the experimental groups and analyzed for the body length and weight, as convenient indicators to determine whether MOMO treatment induced changes in body growth of surviving larvae. Larvae emerged from control food or supplemented with increased concentrations of MOMO had comparable body traits (Figure 9A). In Drosophila food passes sequentially through the foregut, anterior midgut, middle midgut, and posterior midgut, and nutrient absorption takes place along this way in a manner similar to that in humans (Miguel-Aliaga et al., 2018). We thus investigated the oral toxicity of MOMO in surviving larvae assessing common features of apoptotic cell death by TUNEL labeling and cleaved-caspase 3 immunostaining of midgut digestive tract, which corresponds analogously to the small intestine of humans (Buchon et al., 2013; Miguel-Aliaga et al., 2018). As shown in Figure 9B, third instar larvae developed in the presence of MOMO appeared TUNEL-positive. In particular, 100 μg/ml MOMO exposure caused a positive TUNEL staining in the nuclei of midgut cells, demonstrating the occurrence of apoptotic DNA fragmentation. Active caspase 3 was found in the cytoplasm of midgut cells located in the same TUNEL-positive area of the gastrointestinal tract (Figure 9C). Of notice, a dose-dependent increase in apoptosis was observed since the labeling of samples fed with 300 μg/ml MOMO considerably increased.

**Figure 10:**
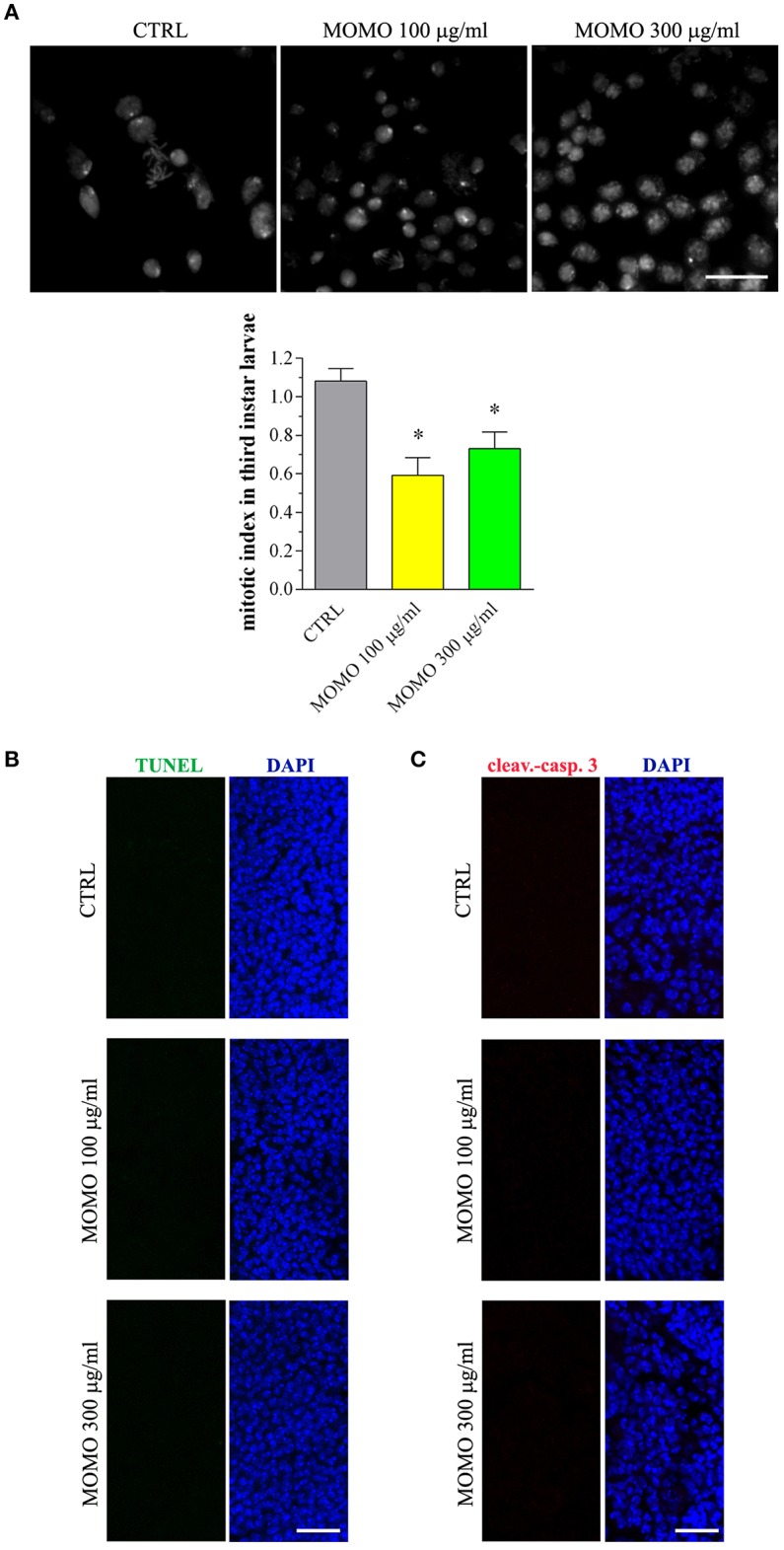
MOMO toxicity in brains of *Drosophila melanogaster*. Third instar larvae emerging at day 5/6 from vials containing control food (CTRL, vehicle) or supplemented with increased concentrations of MOMO were analyzed. **(A)** Mitotic index: images (upper panel) and analysis (lower panel). Scale bar = 100 μm. Results are expressed as fold change of CTRL. ^*^p < 0.01 relative to CTRL. **(B)** TUNEL and **(C)** cleaved-caspase 3 staining of *Drosophila* brains. DAPI (blue) was used for nuclei detection. Scale bar = 20 μm. Images and data are representative of at least 15 larvae collected in 5 independent experiments.

Consistent with a reduced proliferation of neural cells, the brains of surviving larvae fed with increasing concentrations of MOMO had a significant lower mitotic index when compared to control, although the presence of apoptotic phenomena was not detected (Figure 10A). Accordingly, the third instar larval brains developed either in the absence or in the presence of MOMO were TUNEL-negative (Figure 10B). Similar results were obtained with the immunofluorescence analysis of active caspase 3 (Figure 10C).

## Discussion

The drug delivery system based on pH has emerged as an ideal carrier for many drugs, including anti-tumor agents, and is related to the acidic extracellular microenvironment in damaged tissues and some acidic organelles like lysosome and endosome (Swenson, 2016; Sharma et al., 2019). Here we report on the cytotoxic properties of a new small molecule, a synthetic precursor of the natural compound climacostol, which is efficiently activated in mild extracellular acidosis, a common biological consequence of anaerobic metabolism.

### MOMO as a Cytotoxic Prodrug

The results obtained on free-living ciliates demonstrate that MOMO is capable, in mild acidic environment (pH 6.3), to restore the cytotoxic activity of climacostol, whereas no adverse effect is induced by MOM-protected climacostol on ciliates resuspended in physiological buffer (pH 6.8). The substantial stability of MOMO at neutral pH and its easily transformation under acidic conditions in the anti-protozoa cytotoxic climacostol, appears particularly interesting in the light of the potential treatment of some human protozoarian diseases, such as leishmaniasis and malaria, respectively, caused by parasites of genera *Leishmania* and *Plasmodium*. In the case of *Leishmania*, amastigotes live inside the strongly hydrolytic and acid environment (pH around 5.0) of parasitophorous vacuoles (PVs) in macrophages of mammalian hosts (Miguel et al., 2007; Pal et al., 2017). They may represent an ideal target for MOMO that could reach the infected cells as a non-toxic compound, while assuring a safe and exclusive release of active climacostol in PVs. Among the genus *Plasmodium*, the only species capable of producing fatal complications is *P. falciparum*, for which it was reported that development of a high burden of parasites, in slightly acidic conditions, can decrease local extracellular pH until values ranging between 6.2 and 6.4 (Zougbédé et al., 2011). In addition, *in vivo* pH measurements of the parasite digestive vacuole (PDV) resulted in values ranging around 5.2 (Kuhn et al., 2007). Also in this case, the acidic pH revealed both in the extracellular medium surrounding infected red blood cells and in PDV, appears as the ideal chemical trigger for MOMO, so allowing *in situ* releasing of climacostol and avoiding systemic adverse events. It is worthy to note that, if required, the biological activity of the activated MOMO can be easily modulated to induce either a cytostatic or a cytotoxic effect.

The known toxicity of chemotherapy, also on non tumoural cells, often depends on the chemical structure of the used drug which may also be greatly affected by the surrounding environment. In this respect, tumor cell metabolism is fundamentally different from that in normal tissue since active transports and acid-extrusion are important in metabolically active cancer cells, setting the intracellular resting pH by the decrease of intracellular protons (Hulikova et al., 2013). Our study shows the efficient cytotoxic and pro-apoptotic effects of MOMO on mouse melanoma cells. In contrast to the native compound climacostol, MOMO displayed a marked pH-sensitivity since its toxic effects become evident only in mild extracellular acidosis while no detectable actions were achieved in more alkaline culture medium. This indicates that MOMO is chemically tailored in the acidic tumor cell medium to form the effective toxic compound climacostol (Buonanno et al., 2008, 2019; Fiorini et al., 2010; Petrelli et al., 2012; Quassinti et al., 2013; Catalani et al., 2016; Perrotta et al., 2016; Zecchini et al., 2019). Of interest, the pH values inducing MOMO activity are in line with the acidic extracellular pH often measured in both human and rodent *in vivo* tumors, including melanoma xenografts (Wike-Hooley et al., 1984; Tannock and Rotin, 1989; Calcinotto et al., 2012; Chen et al., 2014). A low extracellular pH may decrease the cellular accumulation and activity of weakly basic cytotoxic compounds as chemotherapeutic agents are (Stubbs et al., 2000; Thews et al., 2007, 2014; Viklund et al., 2017), since the passive diffusion of drugs into cells occurs mainly when they are uncharged. pH-responsiveness has always been a hot topic in the controlled drug release field (Sharma et al., 2019). Indeed, there is a general agreement that effective prodrugs should be tailored chemically to become more efficacious in the tumor milieu, as for instance through chemical activation at low pH and/or O_2_ tension (Swietach et al., 2014; Sharma et al., 2019). In this respect, we provide evidence on the very low activity of MOMO, but not climacostol, vs. immortalized mouse cells of non-tumor origin, even in acidic conditions, thus suggesting MOMO being preferentially activated in the unique extra-acidic microenvironment that characterizes tumoural cells. The use of pH-responsive compounds effective in the low-pH tumor microenvironment may represent an effective strategy for lowering the cost of treatment and reducing side effects (Adams et al., 2000; Flowers et al., 2003; Wong et al., 2005; Valiahdi et al., 2013; Swietach et al., 2014; Burns et al., 2016; Wang et al., 2018). We thus identify MOMO as new therapeutic target capable of freeing cytotoxic climacostol more easily in the cancer cells themselves than in normal ones. In this respect, MOM-protection emerges as a potential prodrug strategy which deserves to be further investigated by multiple *in vivo* assays.

The preliminary findings presented here in the model organism Drosophila support the efficient activity and oral delivery of MOMO molecule *in vivo*. Results indicated that MOMO feeding of mating adults affects oviposition. In addition, the eclosion of larvae reared on food containing MOMO was negatively affected. Likely, the MOMO protected molecule is activated by the low pH of food during oral intake, although an additive role of the acidic conditions of the digestive tract cannot be excluded. Reduced survival of *D. melanogaster* was due to lethality during the larval stages while emerging larvae had comparable body traits when compared to control and retained their ability to develop into flying adults. Interestingly, we found that surviving eclosed larvae exhibited an extended damage (cell death by apoptosis) in the gut, further corroborating the notion that orally activated MOMO is consumed and assimilated. Feeding of adult *D. melanogaster* with bleomycin, an anticancer drug that is also widely used as a DNA-damaging agent, caused lethality and enterocyte-specific damage and cell loss in the gut (Amcheslavsky et al., 2009). Because brain tissue of larvae was also affected (reduced mitosis), although, as expected, to a lesser extent when compared to midgut, active MOMO exhibited prolonged toxic effect after oral intake and gut absorption. It has been observed that the first wave of neurogenesis in *D. melanogaster* occurs during embryonic stages, but most of adult brain neurons developed during the larval stages (Homem and Knoblich, 2012; Kohwi and Doe, 2013). In addition, midgut cells have an high proliferating/regeneration rate, even in adult flies (Amcheslavsky et al., 2009; Tian et al., 2017). Accordingly, our data revealed that MOMO efficiently targets different tissues of the developing fly with high metabolic/proliferating activity, such as midgut and brain.

## Conclusions

Currently, a great deal of effort is aimed at discovering novel small molecules or to small molecule derivatives exhibiting the optimum in potency/selectivity, and to exploit new therapeutic indications (Nobili et al., 2009; Gurevich and Gurevich, 2014; Harvey et al., 2015; Dhanak et al., 2017; Cheng et al., 2018; Huck et al., 2018; Schiavone and Trabace, 2018). Living organisms have a high ability to synthesize complex molecular structures with defined biological properties, and the climacostol is a typical example as small molecule (Nobili et al., 2009; Harvey et al., 2015; Catalani et al., 2016). The great advantage in the development of MOM-protecting group in the chemical synthesis of climacostol is not just to provide the best way to solve supply problems, but MOMO can be used as a precursor in biosynthetic transformations. The pH-activated cytotoxic MOMO shows how the synthetic chemistry excels in the invention of unnatural small molecules, another step to use the combination of chemistry and biology in the production of efficient prodrug systems to new applications.

## Data Availability

The raw data supporting the conclusions of this manuscript will be made available by the authors, without undue reservation, to any qualified researcher.

## Author Contributions

ElC: contribution to the experimental plan and analysis work, design and acquisition of mammalian cell and Drosophila data, data processing, and contribution to article writing. RB, SZ, MG, and MC: design and analysis of mammalian cell data. GL: synthesis and chemical characterization of MOMO. CD, CP, and EmC: analysis of mammalian cell data, contribution to article writing. SB and GP: design, acquisition, and analysis of Drosophila data. EM: contribution to the design and conception of the experimental work, synthesis and chemical characterization of MOMO, contribution to article writing. CO and FB: contribution to the design and conception of the experimental work, analysis of protist data, contribution to article writing. AF and SP: contribution to the design and conception of the experimental work, analysis of mammalian cell and Drosophila data. DC: conception and coordination of the whole experimental and analysis work, supervision of all the experiments, analysis/interpretation and processing of data, article writing.

### Conflict of Interest Statement

The authors declare that the research was conducted in the absence of any commercial or financial relationships that could be construed as a potential conflict of interest.
